# Applying the bronchopulmonary dysplasia framework to necrotizing enterocolitis

**DOI:** 10.3389/fped.2024.1388392

**Published:** 2024-05-14

**Authors:** Amy E. O’Connell

**Affiliations:** ^1^ Division of Newborn Medicine, Boston Children's Hospital, Boston, MA, United States; ^2^ Department of Pediatrics, Harvard Medical School, Boston, MA, United States

**Keywords:** NEC, necrotizing enterocolitis, prematurity, intestinal epithelium, development

## Abstract

Necrotizing enterocolitis (NEC) is a devastating disease of the neonatal intestine, causing widespread intestinal necrosis as well systemic illness that frequently results in death. Because the clinical onset of NEC is sudden and difficult to predict, NEC is considered an acute event. However, NEC does not occur *in utero*, meaning that postnatal exposures are required, and it does not typically occur right after birth, suggesting that longitudinal changes may be occurring before NEC can develop. In this perspective, the author considers whether NEC should be re-considered as a problem of disordered intestinal epithelial development, with required maladaptation over time prior to the onset of the necrotic event. This framework is similar to how bronchopulmonary dysplasia is currently conceptualized. They also advocate that NEC researchers incorporate this possibility into future studies on NEC susceptibility and pathogenesis.

Necrotizing enterocolitis (NEC) is an intestinal disease that affects about 7% of all very and extremely premature infants (1). This translates to over 2,500 affected infants annually in the United States, and about one in four of these infants will die from the disease (2). The estimated cost of one case of NEC adds from $75 K–200 K to the cost of a neonatal intensive care unit (NICU) stay (3), and infants who go on to develop short gut syndrome due to loss of bowel from NEC have five year costs that total well over $1M (4). NEC can also lead to poor neurodevelopmental outcomes, and NEC requiring surgical intervention is independently associated with long term neurodevelopmental impairment (5). NEC causes intestinal inflammation and necrosis of the bowel, and while much progress has been made in understanding what NEC actually is in the past ten years, we don't fully understand why it occurs and we have limited strategies to prevent or treat NEC (6). Older infants may occasionally get NEC, but evidence suggests that the pathophysiologic mechanisms that cause NEC in older infants are distinct from classic, prematurity-associated NEC (7, 8). Certainly, NEC is not a clinical entity that occurs in older children or adults. The specific predilection of NEC to occur most commonly around 30 weeks' postmenstrual age suggests that there are developmental phenomena occurring around this period that predispose the intestine to NEC.

Key developmental phenomena are in flux in the intestine during weeks 25–35 of gestation. While intestinal crypts are already developed, they are immature and shallow in the last few weeks of the second trimester and expand dramatically in the next three months. In addition, Paneth cells, which provide not only critical antimicrobial peptides but also stem cell supporting factors like Notch ligands and Wnt ligands, are present early in development and expressing antimicrobial proteins, but their granules only become histologically apparent around 30 weeks, suggesting that their granules may not be fully matured or capable of releasing proteins in earlier stages (9). Steven McElroy's group has highlighted the developmental role of Paneth cells by showing that, once Paneth cells come on the scene in mouse models, you can no longer induce NEC without first depleting them (10). We have some knowledge of these key changes but in general this time period is understudied due to the inherent difficulties in obtaining healthy tissues from premature babies to study normal development. Much of what we know is from patients with atretic tissues that were resected or subjects who already had NEC, and from recent RNA-based single cell sequencing studies in fetal tissues, which may not be reflective of protein production or cellular function (9, 11).

While NEC has peak incidence in premature infants around 30 weeks' postmenstrual age (8), NEC does not occur *in utero* at 30 weeks of pregnancy (or, for that matter, at any time *in utero*). This highlights the role of postnatal exposures in the development of NEC. In fact, mouse models of NEC rely on postnatal exposures to trigger NEC, including but not limited to formula feeding, microbiome changes, and hypoxia (12). These triggers have been associated with the development of NEC in premature babies, but no one etiology is a definitive, reliable trigger of NEC. Many groups have identified defects in the immune axis including components of immature immune function and disordered microbiome composition that may contribute to NEC pathogenesis (13–16), however, these exposures do not immediately and uniformly trigger NEC in human babies.

The broad array of factors that have been shown to enhance NEC susceptibility suggest that it is not the particular insult that is critical, but perhaps the impact it has on the developing intestine. We tend to think of NEC as an abrupt process, primarily because it appears to happen suddenly and is typically rapid in progression once it is diagnosed. However, most neonates who develop NEC do not do so immediately after birth or after immediately beginning enteral feeding (7). There is a postnatal lead time that is needed before NEC can occur. The third trimester is a critical time in continuing intestinal growth and maturation. The small and large intestine are not only continuing to lengthen, but the villi are expanding in length and number, crypt fission is ongoing, and intestinal crypts are continuing to lengthen and mature in their composition (17–19). On a cellular level, not only are the Paneth cells continuing to functionally mature, but the intestinal stem cell niche is maturing and the numbers and types of epithelial secretory and absorptive cells is continuing to expand (20). In the intestinal submucosa, important mesenchymal cell types and smooth muscle cells that are critical for supporting the ISC niche and protecting the submucosal space from the intestinal lumen are also growing and maturing (17), as is the mucosal immune system (21–23). There are likely to be important changes in microvasculature and the lymphatic lacteals in the villi that are still occurring as well, but this is not extensively studied.

It may be worth taking some points from our colleagues studying bronchopulmonary dysplasia (BPD) as we consider the sequence of events occurring in NEC. In BPD, we know that the premature respiratory epithelium is immature, and that *ex utero* exposure to higher oxygen tension than the infant would see *in utero* leads to disordered development of the respiratory epithelium. This is characterized by tissue remodeling changes that result in emphysema and increased fibrosis in addition to cellular changes that include impaired function of respiratory stem cells (24). The premature gut is also quite underdeveloped in contrast to a term infant, however. While we do not histologically define the stages of the intestine in the same way as the lung (embryonic, pseudoglandular, canalicular, saccular, alveolar), one could envision a similar theoretical schema highlighting the different phases of fetal intestinal development where a 30-week fetus is quite immature compared to a 40-week one (Figure 1). In the time a premature infant spends exposed to the non-uterine environment before getting NEC, it is possible, and rather likely, that the normal development of the intestine is being impacted by all of the additional stimulation and the functionality now being required of it. This could lead to diminished digestion, impaired mucosal immunity, abnormal enteroendocrine cell responses, and regional blood flow alterations. And as we have learned about BPD, the same *ex utero* exposures impede or alter epithelial maturation in some infants more than others. This may be where genetic propensities come into play in increasing NEC susceptibility, as polymorphisms that lead to increased maladaptation to the *ex utero* exposures may enhance NEC risk. In this framework, for NEC to occur three things have to happen: exposure to *ex utero* life, a maladaptive epithelial response to these exposures, and finally an accelerant insult that pushes the altered epithelium toward necrosis (Figure 2).

**Figure 1:**
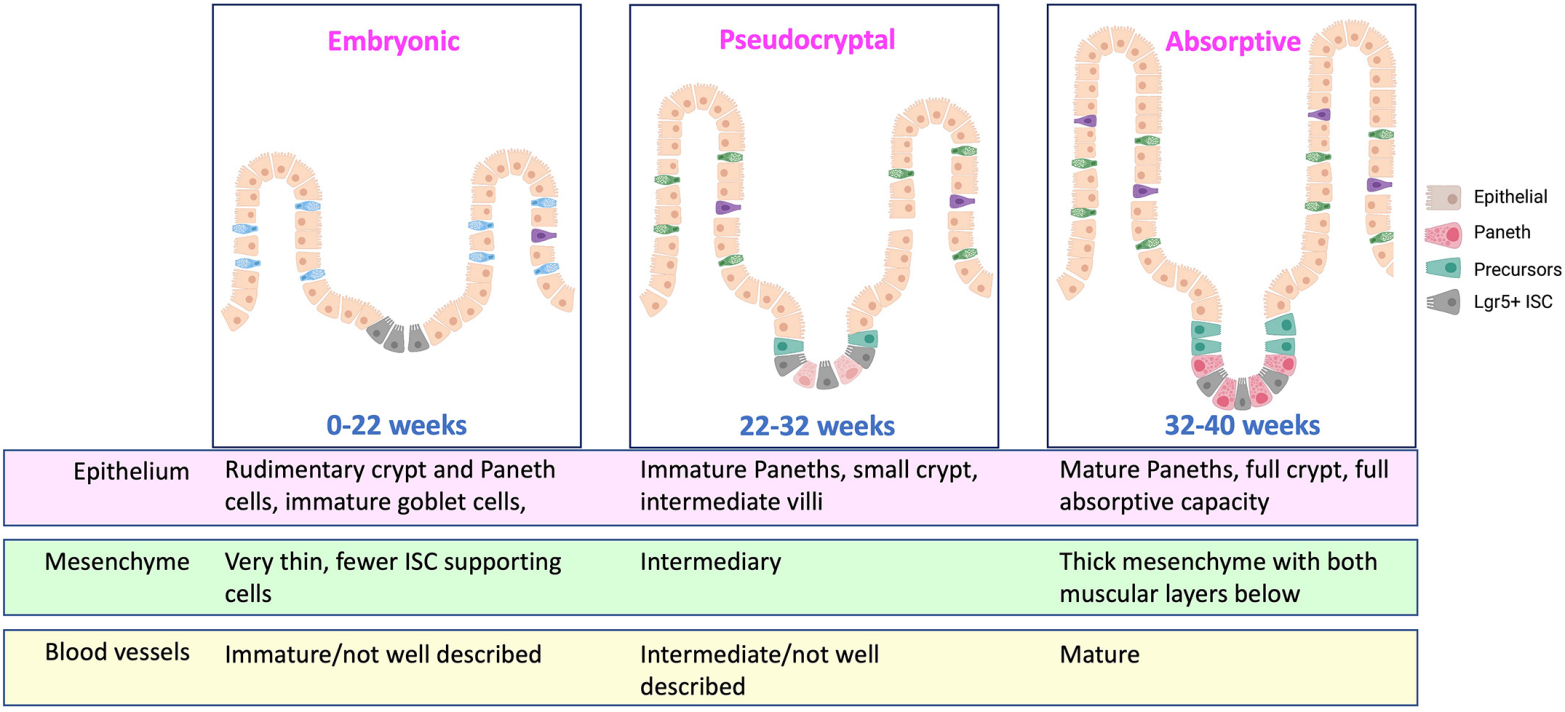
Schematic of developmental stages of the intestine during prematurity. Currently known important developmental alterations are detailed. Approximate age ranges are estimates and additional important distinctive phases may be present, but this developmental period is understudied in humans, and additional information is needed. Created with Biorender.com.

**Figure 2:**
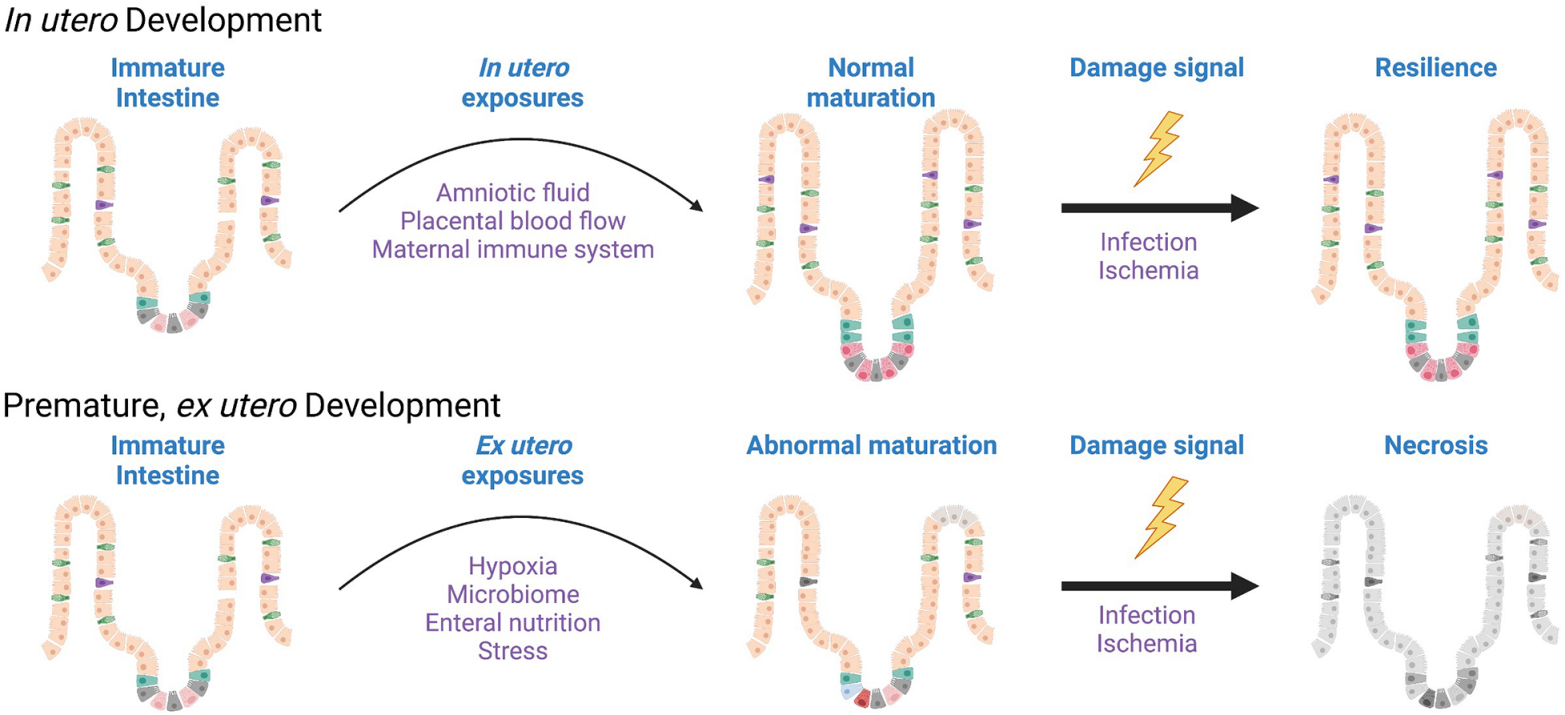
Disordered development of the epithelium is a component of necrotizing enterocolitis. While NEC occurs clinically as an acute insult, the postnatal remodeling of the immature intestinal milieu, and the intestinal epithelium in particular, are required in order for NEC to manifest. Characterization of these *ex utero* developmental changes would inform preventative strategies. Created with Biorender.com.

In order to think of new ways to prevent NEC, we need to think about the pre-NEC events that happen in the disordered development stage. These factors are challenging to study due to a lack of models that recapitulate human development. For example, in rodent models of NEC, much of the intestinal development that normally occurs *in utero* in humans happens postnatally. Intestinal development in humans happens at a time when humans are not yet designed to be encountering a full microbiome or atmospheric oxygen tension, while in rodents this is status quo. Pigs and non-human primates have a more similar intestinal development schedule to humans but are cumbersome for different logistical (and ethical) reasons (12). Human intestinal organoid modeling has exciting potential for studying adaptive responses of the developing epithelium to postnatal exposures, and the culture systems can be manipulated to recreate a variety of *in vivo* exposures including solute load, microbiome, and oxygen tension. Potential refinements and improvements in human organoid modelling such as adding a comprehensive immune system, creating a microvasculature with functional blood supply, and/or integrating the enteric nervous system would further enhance our ability to understand the postnatal adaptations occurring after premature birth.

In conclusion, while most of the research focus on NEC centers around the acute process of immune-mediated necrosis, additional attention should be given to the postnatal adaptations of the intestinal epithelium that may precede the immune-mediated finale. I propose that we begin to consider more thoroughly how postnatal adaptations of the immature intestinal epithelium may create the necessary environment for the downstream tissue injury. Identifying opportunities to modulate these precursor changes may ultimately reveal preventative strategies to stop this deadly disease.

## Data Availability

The original contributions presented in the study are included in the article/Supplementary Material, further inquiries can be directed to the corresponding author.
